# Cyclic Behavior of Cellular Glass Aggregates: An Experimental Comparison with Natural Aggregates

**DOI:** 10.3390/ma19050993

**Published:** 2026-03-04

**Authors:** Layal Jradi, Bassel Seif El Dine, Jean-Claude Dupla, Jean Canou

**Affiliations:** 1 College of Sciences and Human Studies, Prince Mohammad Bin Fahd University, Dhahran 34754, Saudi Arabia; bseifeldine@pmu.edu.sa; 2 Ecole des Ponts ParisTech, Champs sur Marne, CEDEX 2, 77455 Marne la Vallee, France; jean-claude.dupla@enpc.fr (J.-C.D.);

**Keywords:** cellular glass aggregates, cyclic behavior, large-scale triaxial, recycled glass

## Abstract

The construction sector is a major user of natural materials and a key contributor to global carbon emissions. To tackle these environmental challenges, the use of recycled products has become increasingly important in modern engineering. Cellular glass aggregate (CGA), made from recycled glass, is a material with potential as a sustainable alternative to natural aggregates. This study characterizes the cyclic behavior of CGA using a large-scale triaxial apparatus, focusing on seismic-relevant properties such as the damping ratio and Young’s modulus. Local displacement transducers (LDTs) were implemented to improve measurement at small strains. The results show that CGA exhibits strain-dependent stiffness and damping behavior comparable to natural aggregates at moderate strains (10^−4^–10^−3^). The Young’s modulus ranges from approximately 300 to 600 MPa, while damping ratios remain at approximately 2–3% for low values of strains (10^−5^). As strain increases to moderate levels (10^−4^–10^−3^), the Young’s modulus decreases to approximately 80–250 MPa, accompanied by an increase in damping ratio to approximately 4–6%. At higher strain levels ≥ 10^−3^, the Young’s modulus further reduces to approximately 40–80 MPa, while damping ratios increase to approximately 7–10%**.** These stiffness degradation and damping trends fall within the ranges reported for sands and gravelly soils in the literature, indicating that CGA can reproduce the cyclic mechanical behavior of natural aggregates under well-defined strain conditions.

## 1. Introduction

Growing environmental problems, high carbon emissions, and the depletion of natural resources have increased the demand for sustainable materials across various sectors. In the engineering sector, there is a strong demand to reduce environmental impact by utilizing eco-friendly and resource-efficient products. For this purpose, the use of different types of recycled products in civil engineering and construction has become an important topic of extensive research [1,2,3,4]. Glass is one of the few products that can be recycled repeatedly without losing its quality and purity [5]. However, current studies prove that worldwide, only 21% of the total volume of glass produced is being recycled [6]. This huge amount of waste glass raises the need for further research to explore new sustainable and high-performance construction materials that are capable of reducing environmental burdens while improving engineering performance. Among these emerging materials are cellular glass aggregates (CGAs), also known as foam-glass. This material has been a focus of broad research, as it has a number of interesting properties like low density (one-tenth the density of gravel), self-stability, and high drainage. Because of this, they have been used extensively in various projects like expanded polystyrene materials, cellular cemented clays, and lightweight concrete [7,8,9,10,11,12,13]. Produced by foaming finely ground recycled glass at very high temperatures reaching 900 °C, followed by subsequent cooling and fracturing of the foam mixture, CGA provides a lightweight granular material where its density usually ranges between 100 and 200 kg/m^3^ [14,15,16,17]. The density of the product aggregate is very low compared to the natural particles, resulting in the low density of the mixture as well as other properties like cooling and sound absorption abilities of the mixture [18,19,20]. The designed porosity of the material also further boosts the resistance of the material to the freeze–thaw action, hence its suitability in a broader range of environmental conditions [21]. The use of this material could help reduce the need for natural aggregates, and consequently, it reduces the need for mining and quarrying activities that usually consume large amounts of energy, disturb ecosystems, and contribute to habitat damage. Recent research has demonstrated that recycled glass materials have potential use as a construction material in various geotechnical and pavement works. The studies demonstrated that CGA is applicable as a construction material in the production of embankments and pavement sub-bases [22,23,24]. CGA has been investigated and approved for a variety of structural and insulation-related applications [25,26,27] as well as fibers for improving problematic soils [28,29] and manufacturing ceramics [27,30]. Cellular glass aggregate is now reasonably well characterized as a crushable, lightweight granular fill whose static compressibility, shear strength, and cyclic stiffness—strongly controlled by density/compaction and particle breakage—have been quantified to support embankment and lightweight backfill design [3,13]. However, there is inadequate information about the mechanical characterizations of this material, especially for small strain values [31,32,33]. There is now a cohesive but still incomplete body of work that links the physical attributes (unit weight, gradation, porosity, and particle condition) and mechanical responses (static compressibility, shear strength, dynamic modulus/damping, and breakage) to performance in geotechnical systems—primarily lightweight fills, embankments, and reinforced backfills, with emerging evidence for pavement-type cyclic performance [13,34,35]. The most robustly supported application space is lightweight backfills and embankments, where unit weight reduction and shear strength/interface behavior are well documented [13,22,35,36]. Pavement and railway layer performance (resilient modulus, cumulative deformation, and response under very small strains) is recognized as important and has been partially studied, but the database is smaller and more fragmented [13,34,36,37,38]. With all these considerations, the purpose of this research is to investigate the behavior of CGA with the use of large triaxial equipment due to the large size of the glass aggregates, for which this test cannot be done with the classical equipment for triaxial tests. A series of cyclic tests have been performed for small values of strain in order to expand the knowledge on cellular glass aggregates and to assess their engineering performance and applicability in modern civil engineering. This study also seeks to present a comprehensive understanding of CGA behavior and compare it to that of natural aggregates, notably in terms of its damping ratio and Young’s modulus, which are two important parameters for the assessment of its cyclic behavior and evaluation of its potential as a sustainable alternative.

## 2. Materials, Testing Setup, and Experimental Procedure

### 2.1. Materials

**Figure 1:**
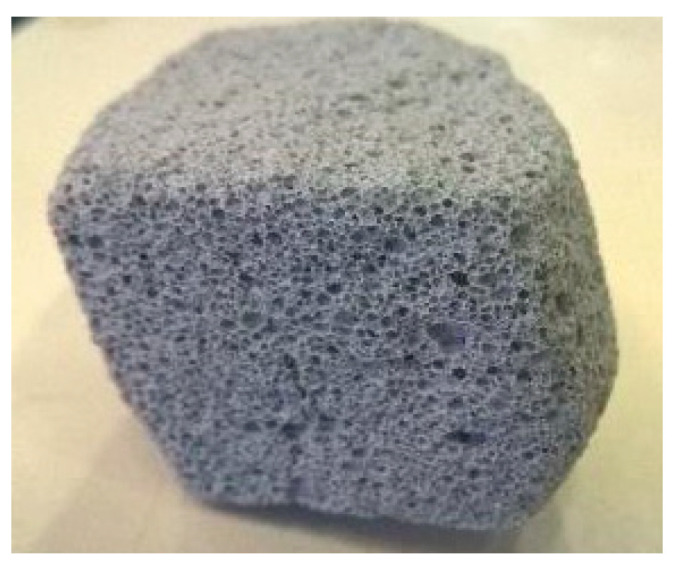
Cellular glass aggregate.

The photo of CGA used in this research work is shown in Figure 1. This material was imported from Misapor, Switzerland. Unlike mechanically crushed natural aggregates, the CGA particles appear sub-rounded to rounded with limited angularity. This morphology can reduce the degree of binder–aggregate interlock when compared to natural aggregates, potentially affecting its shear resistance and formulation of granular mixes [39].

#### 2.1.1. Definition of the Weathered Material

**Figure 2:**
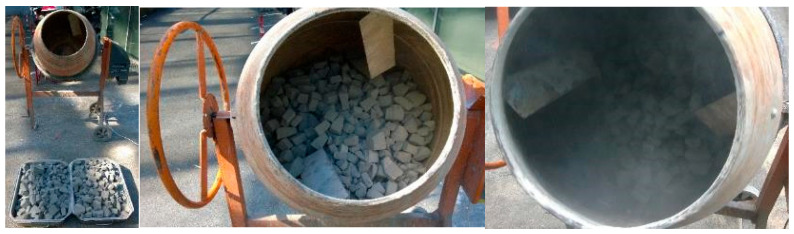
Weathering procedure of the material.

During handling and transportation, the material might undergo several stages that might cause damage. To examine this effect, a weathering process was carried out on the material by mechanically agitating it using a cement mixer. The intact material filled the cement mixer to 45% of its capacity, which in this case carried a volume of 61 L and a weight of 10.8 kg. The agitation ran for 2 min while setting the mixer at an angle of 45° relative to the horizontal position. This step was done as shown in Figure 2 below.

The weathered material was gathered and sieved to study the effect of the weathering procedure on the material. the grading of the whole and weathered aggregate revealedthere was a fair reduction in the larger particles, as indicated by the reduction from 21% to 15% alongside the increase of 4% in particles that are smaller than 31.5 mm.

#### 2.1.2. Definition of the Compacted Material

Compression of the material would be necessary for CGA-related industrial applications. Therefore, it is crucial to describe and measure the impact of the compaction process.

**Figure 3:**
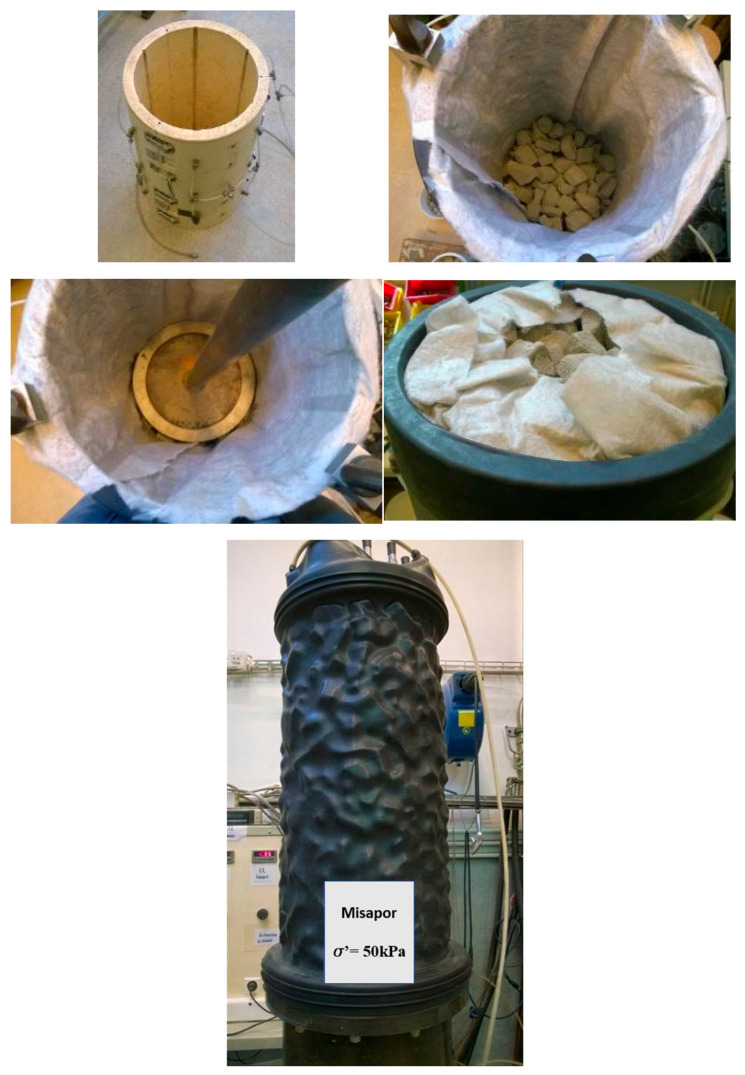
Reconstitution of the material specimen.

To develop a controlled test and determine how this test will affect the material’s degradation, a number of compaction tests were carried out. The test was carried out using two dry densities, ρd = 230 kg/m^3^ and ρd = 212 kg/m^3^, corresponding to compaction ratios (relative to the natural state) of 1.29 and 1.19, respectively. Since the densities were known as well as the volume of the mold, which is 0.0424 m^3^, the masses of material required to produce specimens with ρd = 212 kg/m^3^ and ρd = 230 kg/m^3^ were computed and found to be 9 kg and 9.75 kg, respectively. The material is then divided into six equal quantities in order to ensure good compactness all over the mold. Then, each layer of the material was compacted to ensure a thickness of 10 cm, which resulted in an overall thickness of 60 cm, which corresponds to the exact height of the triaxial mold. The compaction of each of these layers was done with the help of a PVC disk. The compaction was done manually by dropping the compaction tool from a fixed height against the plastic plate holding the compacted PVC mixture. The reconstitution process of the specimens is shown in Figure 3.

### 2.2. Testing Setup

**Figure 4:**
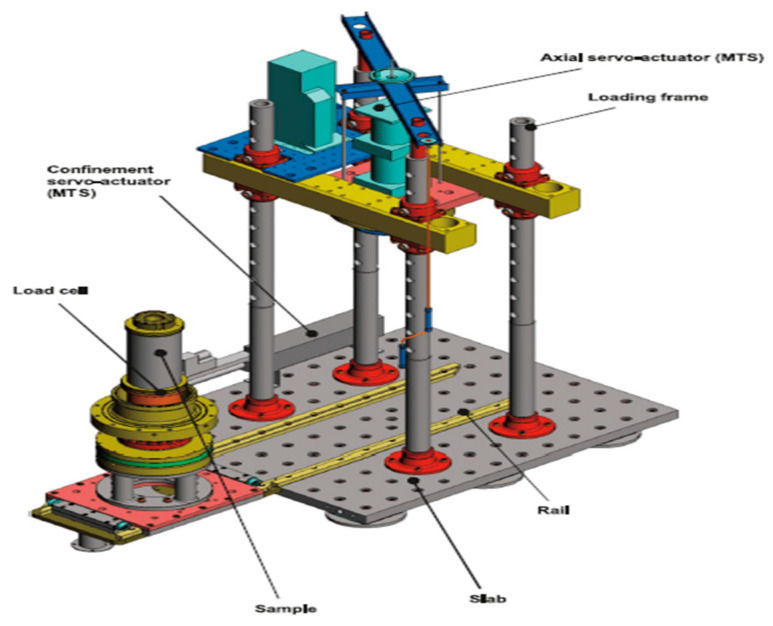
Triaxial testing device.

In this work, the cyclic response of CGA is studied based on a sophisticated large-scale triaxial testing system designed for shearing large-sized soil specimens. The apparatus permits monotonic and cyclic loading to be applied, and the confining pressure, level of deformation, frequency of loading, and saturation state to be independently controlled in order to closely replicate typical in situ conditions. Cylindrical specimens of diameter 300 mm and height 600 mm were used for the test. The experimental setup is composed of a large triaxial chamber that is mounted in a four-column load frame and is equipped with a hydraulic actuator that can provide axial loads up to 500 kN. Confining pressure in the chamber is controlled by another hydraulic actuator. Sample preparation is done outside of the loading frame, and then the triaxial cell is set up under the actuator for the test. An overview of the testing system is presented in Figure 4.

Before evaluating the behavior of the material, an initial conditioning phase was applied in order to eliminate the permanent deformations. This phase is a deviator-controlled phase that consists of applying 20,000 cycles at a frequency of 1 Hz and at a given level of deviatoric stress. Following this conditioning phase, cyclic stress-controlled loading is applied to the specimen by using two levels of isotropic consolidation stress (σ’c), namely 100 kPa and 50 kPa, combined with variable deviatoric stress amplitude. For each combination of stresses, 100 loading cycles are applied. A brief summary of the experimental program is provided in Table 1.

## 3. Results

Test C7 was the first test for which the local measurement of deformations has been properly implemented.

### 3.1. Accommodation Phase (Test C7)

**Figure 5:**
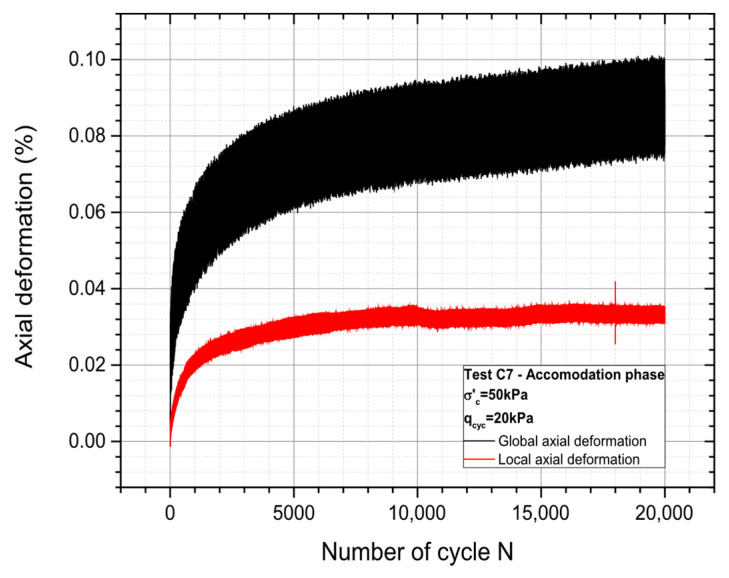
The material’s axial deformation during the accommodation stage.

20,000 deviator-controlled cycles have been applied to accommodate the material, with a constant deviator amplitude Δq_cyc_ equal to 20 kPa (frequency of 1 Hz). Both global and local deformation measurements have been made. The local strain is assessed by a Local Deformation Transducer (LDT) designed by the well-known researchers Goto et al. (1991) [40]; in this case, the LDT is capable of measuring the mean value for the local strain of the soil samples subjected to shearing in the laboratory. The overall accuracy of the strain measurement is approximately 10^−6^ [34]. Figure 5 presents the corresponding results. As expected, the local measurements provide lower values of the deformations with respect to the global measurements, which is a typical result. Local measurements are more realistic (and reliable) and should preferably be considered for further calculations. Another important point is that there is, after 20,000 cycles, a clear stabilization of the deformation in terms of local measurement, whereas the global deformation continues to slightly increase.

Six displacement-controlled cyclic loading sequences have been implemented in that test, as described in Table 1. These sequences were designed to progressively investigate the material response under increasing axial strain amplitudes while remaining within a non-destructive regime.

**Figure 6:**
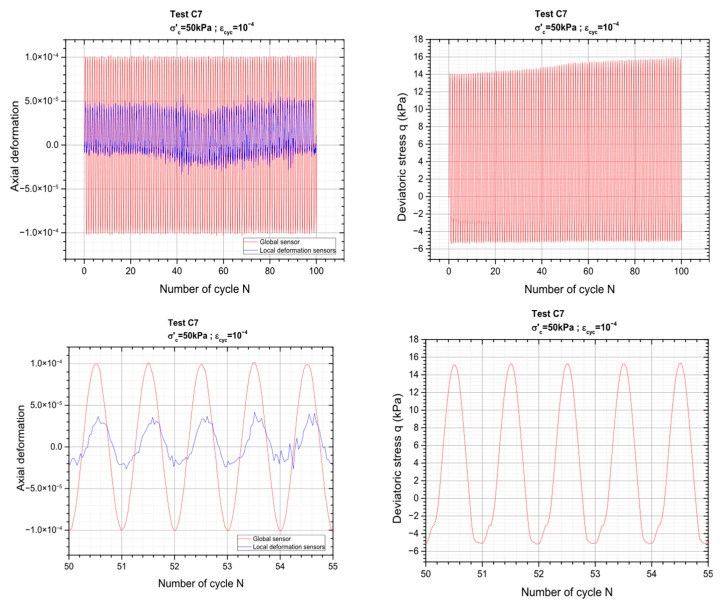
Detailed view of the loading applied and material response during a cyclic sequence of test C7.

Figure 6 below presents typical results obtained for the order sequence that corresponds to Δε_cyc_ equal to ±10^−4^.

Again, a good consistency is found between global and local measurements throughout the loading cycles. As expected, local measurements exhibit slightly smaller strain amplitudes compared to global measurements, which can be attributed to strain localization effects and differences in gauge length and measurement techniques. Both signals are clearly in phase, indicating a coherent material response and confirming the reliability of the instrument. The slight offset visible on the zoom is only due to a slightly different time origin taken for both types of measurements.

**Figure 7:**
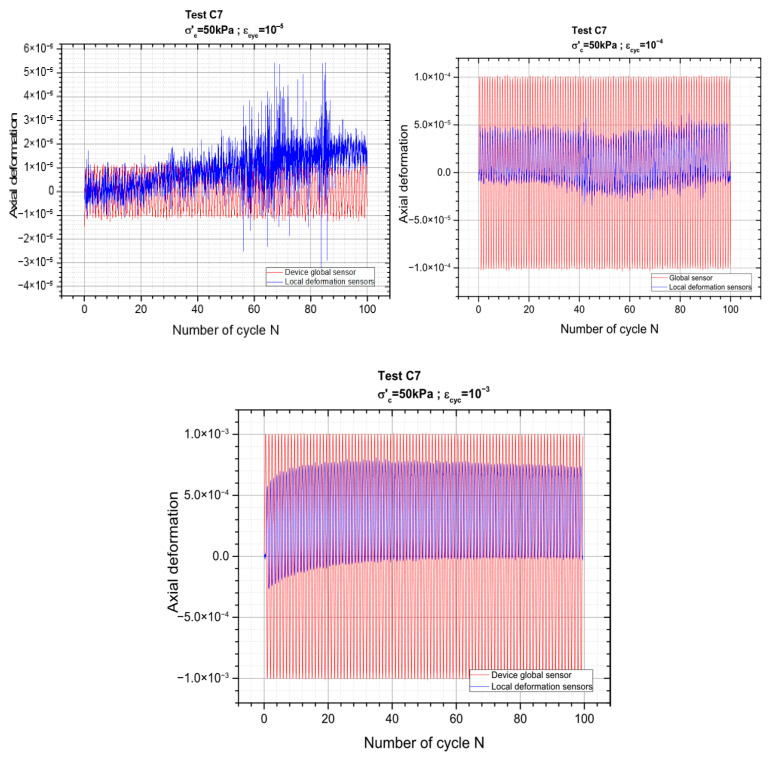
Cyclic loading applied: global and local deformations.

Figure 7 below presents the results corresponding to the 3 successive cyclic sequences carried out for axial deformation amplitudes (ε_cyc_) of 10^−5^, 10^−4^ and 10^−3^. The results for the other three levels of deformations can be found in the Supplementary Material. The gradual increase in deformation amplitude allows for a systematic assessment of the material behavior across a wide strain range while minimizing damage accumulation.

As the strain amplitude increases, the material response becomes progressively more pronounced, with clearer stress–strain loops.

**Figure 8:**
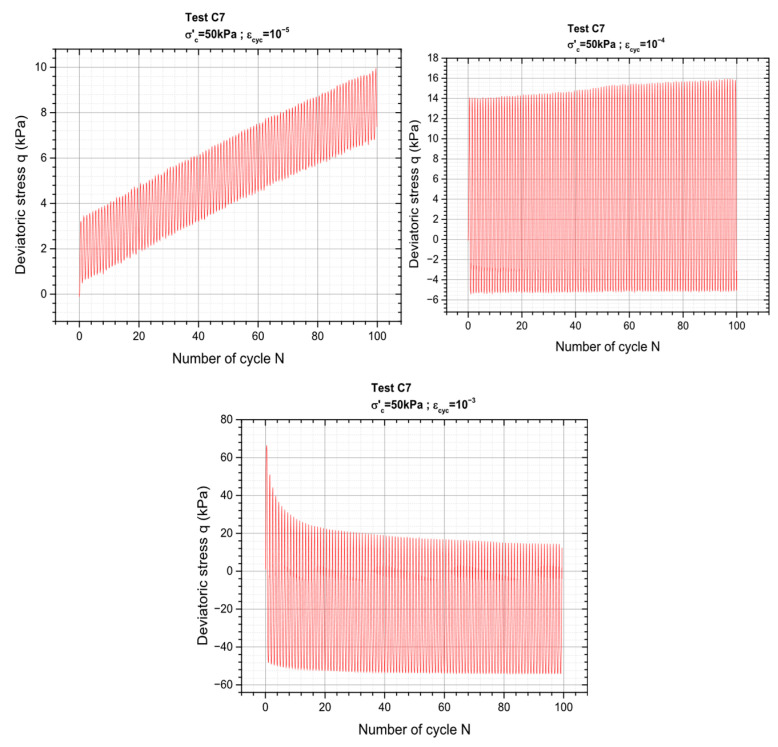
Material behavior under deformation-controlled repeated loading.

Figure 8 below presents the results of the test in terms of material response for three levels of deformation where the variation of the deviatoric stress as a function of number of cycles is plotted. The rest can be found in the Supplementary Material.

The purpose of the cyclic testing was to investigate the behavior of the material under various and controlled loading with a low amplitude within a short number of cycles. The specimens were subjected to successive sequences with gradually increasing amplitudes. This approach enables the evaluation of stiffness degradation and energy dissipation characteristics without inducing irreversible damage. Since the tests were conducted within a non-destructive regime, they provide a suitable framework for monitoring changes in important mechanical parameters such as damping ratio and secant modulus.

**Figure 9:**
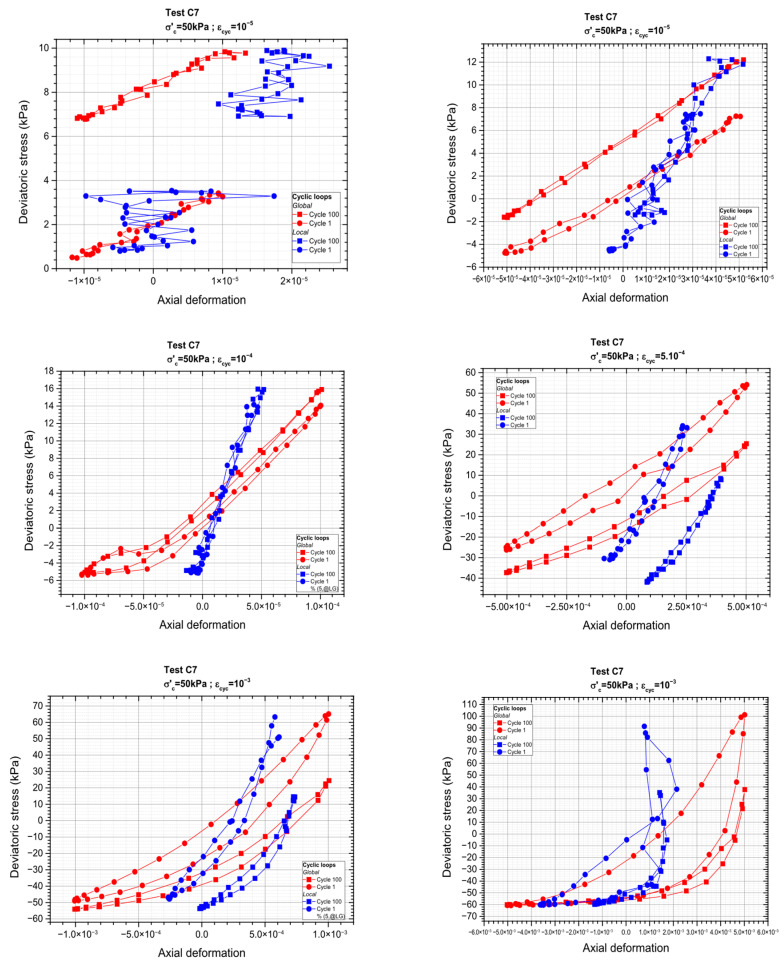
Cyclic load loops (cycle n°1 and cycle n°100).

Figure 9 below illustrates the obtained outcomes in terms of stress–strain loops for the first and the 100th cycle, for each cyclic sequence. These loops provide insight into the evolution of stiffness and energy dissipation over repeated loading. The first level is poorly defined, which makes it an unreliable reference point for analysis since its boundaries are unclear. Analysis of the results shown in Figure 8 corresponding to the first three deformation levels ε_cyc_ = 10^−4^, ε_cyc_ = 5 × 10^−5^, and ε_cyc_ = 10^−5^ indicates that hysteresis loops at very low strain levels are more difficult to define precisely. This is primarily due to the extremely small displacement amplitudes associated with this strain level, which approach the limits of measurement resolution. As a result, the resulting loops appear less well-defined and exhibit greater uncertainty compared with those obtained at higher deformation levels.

**Figure 10:**
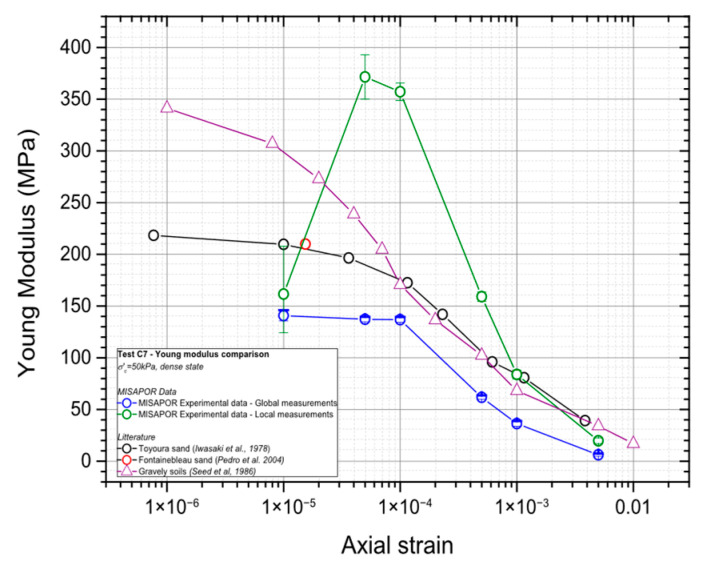
Variation in the Young’s modulus of CGA with the cyclic deformation level compared with sands [41,42], and gravelly soils [43].

The damping ratio and Young’s modulus were determined using the stress–strain loop from the 100th cycle at each tested deformation level. Figure 10 shows the deformation level plotted versus the Young’s modulus, together with usual values for sands and gravelly soils. The comparison is made using values from the literature for different types of gravel and sand. This contextualization helps highlight how the tested material behaves relative to well-documented granular soils and provides a clearer understanding of its stiffness characteristics across different strain ranges.

**Figure 11:**
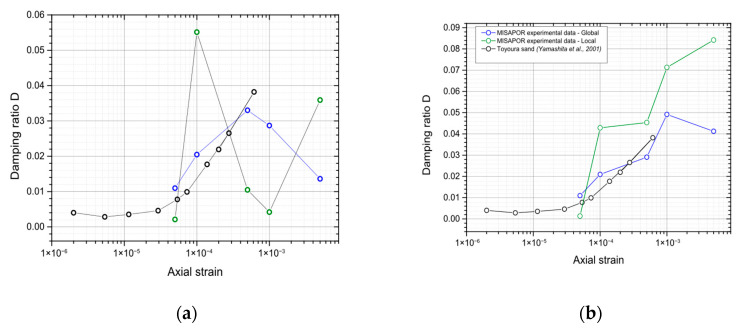
Changes in the CGA damping ratio in relation to the degree of cyclic deformation in comparison to Toyoura sand [44]: (**a**) raw data; (**b**) estimation of the elastic energy based on the Young’s modulus.

Figure 11 presents the results obtained for the damping ratio. Figure 11b was plotted by estimating the elastic energy of each hysteresis loop using the measured Young’s modulus. For the first levels, the results derived from the raw data and those obtained using the elastic energy are generally consistent, indicating reliable loop definition. However, for the very small deformations where the loops become less clearly defined, the two approaches begin to diverge, reflecting the growing uncertainty in the energy calculation and loop interpretation.

**Figure 12:**
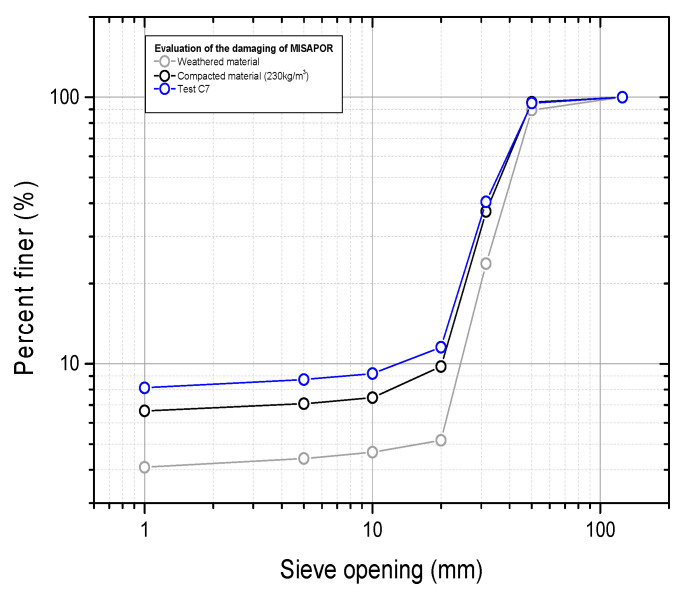
Assessment of the material degradation induced by the cyclic test.

Figure 12 below presents the variation in the grading curve of the material collected after the test with respect to both weathered material and compacted material. The grading curve shows a very small difference with respect to the compacted material, especially in the fine particles, where this can be attributed to more crushing of medium-fine particles.

### 3.2. Presentation of Test C14

Test C14 has been carried out under similar conditions and similar protocol as test C7, but for an isotropic consolidation stress of 100 kPa. This made it possible to assess the effect of this parameter on the obtained measurements.

#### 3.2.1. Accommodation Phase

**Figure 13:**
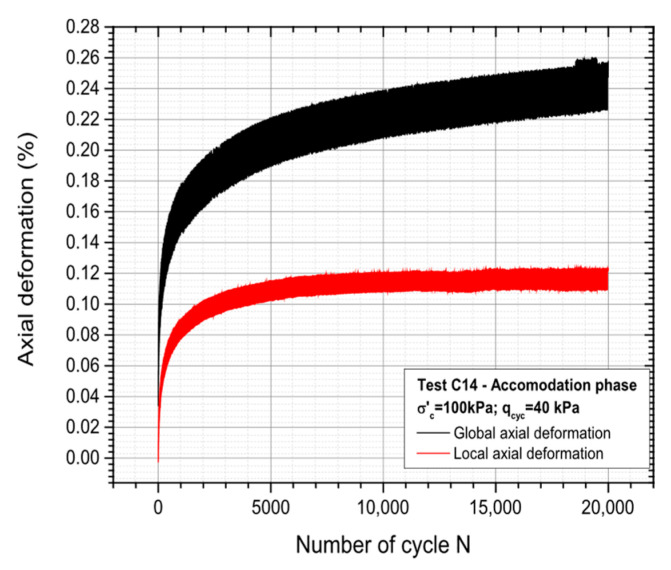
Global and local axial deformation of the sample during the accommodation stage.

20,000 stress-controlled cycles (Δq_cyc_ = 40 kPa) have been applied to the specimen for accommodation. Figure 13 shows the obtained results in terms of global and local strains. Similar comments as the ones done for test C7 may be done, with a clear accommodation observed in terms of local strain, whereas the global strain still keeps increasing slightly.

#### 3.2.2. Displacement-Controlled Loading Sequences

**Figure 14:**
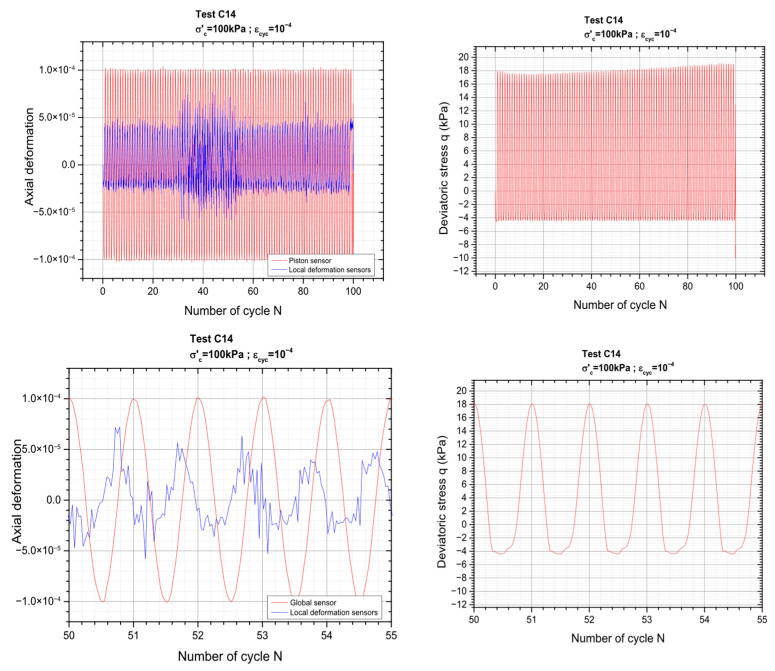
Close-up view of the loading signal and material behavior during a cyclic sequence of test C14.

**Figure 15:**
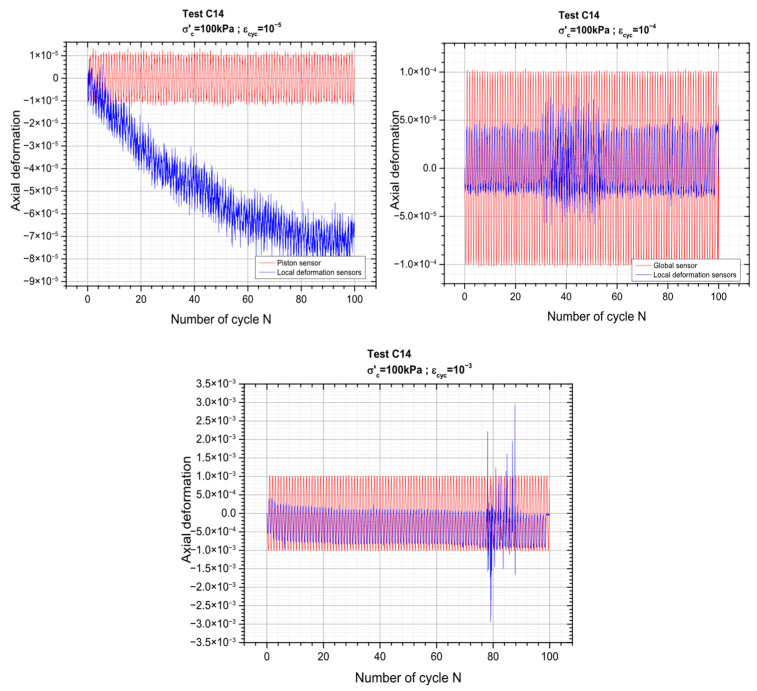
Cyclic loading sequences: global and local measurements.

Loading sequence corresponding to a cyclic axial deformation of 10^−4^ is schematically depicted in Figure 14 below, while the results for the remaining values of axial deformation corresponding to the other values of axial deformations (ranging between 10^−5^ and 10^−3^) are shown in Figure 15.

**Figure 16:**
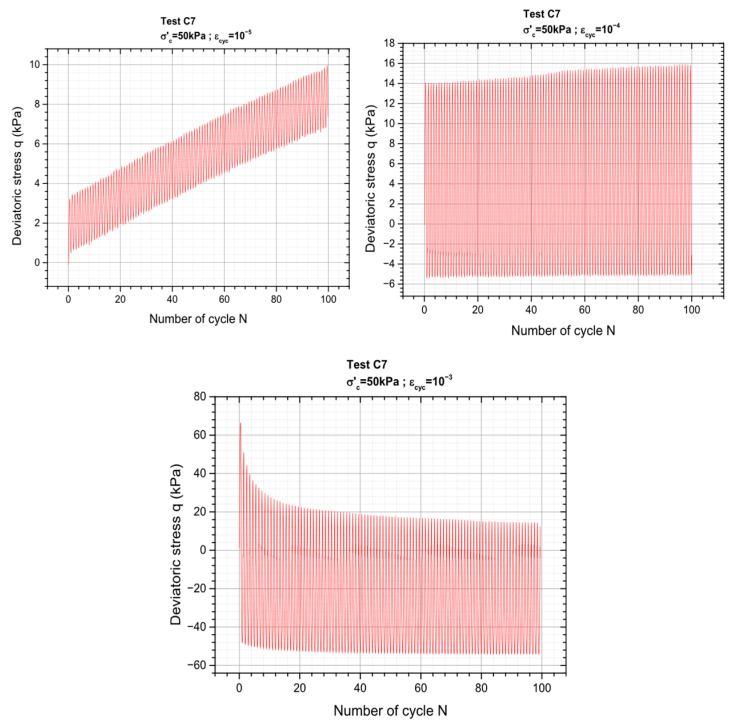
CGA reaction to cyclic load sequences controlled by deformation.

Figure 16 shows the variation of the deviatoric stress as a function of the number of cycles for three series of axial deformation.

**Figure 17:**
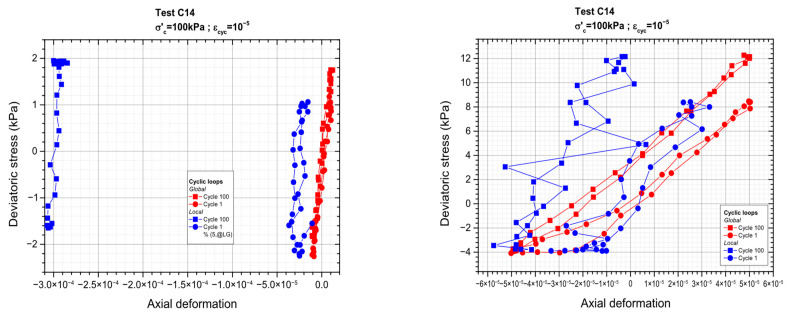

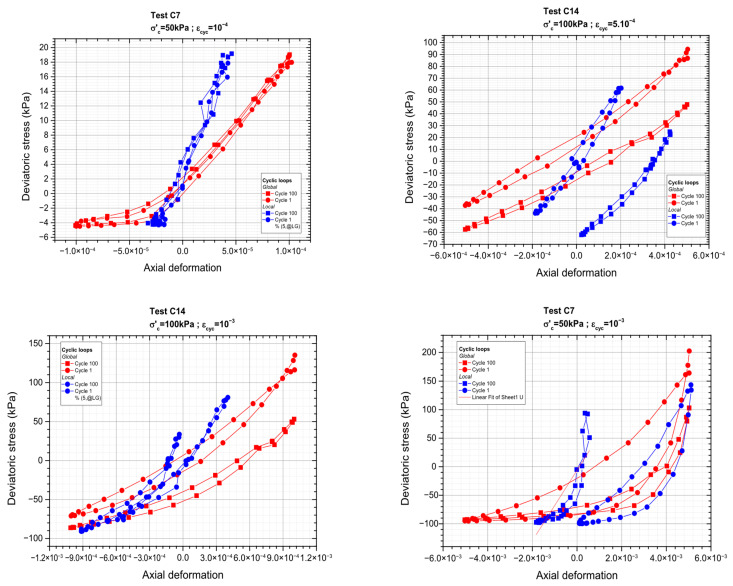
Cyclic stress–strain loops.

Figure 17 below shows the cyclic results presented through the stress–strain diagram for the first and 100th cycles for each cyclic test. The same thing has been seen for test C7, where for the strain levels indicating the first three cycles, εcyc = 10^−4^, εcyc = 5 × 10^−5^, and εcyc = 10^−5^, the diagram is not visible, hence not a recognizable point for analysis. This is due to the low displacement amplitudes associated with this strain value.

**Figure 18:**
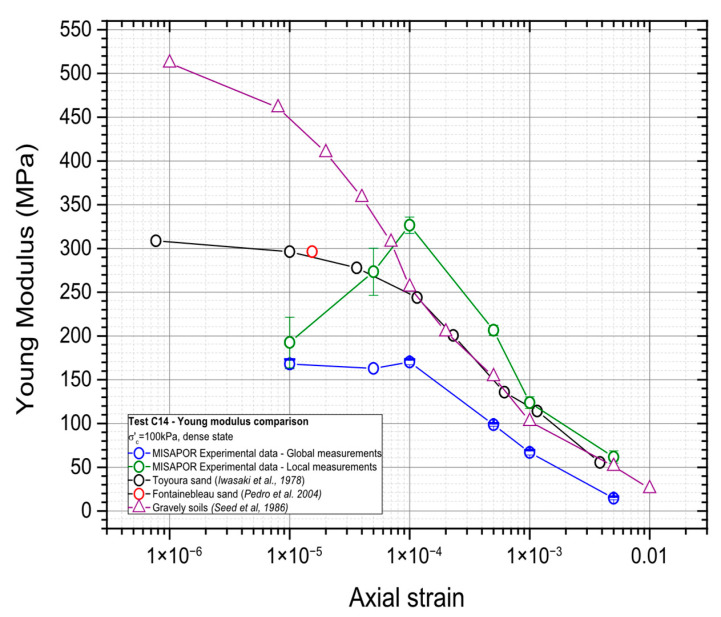
The Young’s modulus of CGA varies according to the degree of cyclic deformation and is compared to the behavior of sands [41,42], and severely deformed soils [43].

The values of Young’s modulus were determined from the stress–strain hysteresis loop corresponding to the 100th cycle of stress and strain values. The results of the Young’s modulus are shown in Figure 18 along with values for different granular materials reported in the literature.

The results show very good consistency for higher strains and the measured modulus values closely follow the trends observed for comparable granular materials. Whereas for very small strains the curves seem to diverge, this might be due to the difficulty in accurately defining the stress–strain loops at these levels of strain, and consequently, the assessment of the Young’s modulus was not precise. Overall, the CGA material shows a strong similarity to gravelly soils, particularly at moderate to high strain levels, where the Young’s modulus values are nearly identical. the discrepancies observed at smaller strains are not considered representative of intrinsic material behavior but are instead linked to measurement uncertainty arising from poorly defined loops in this strain range.

**Figure 19:**
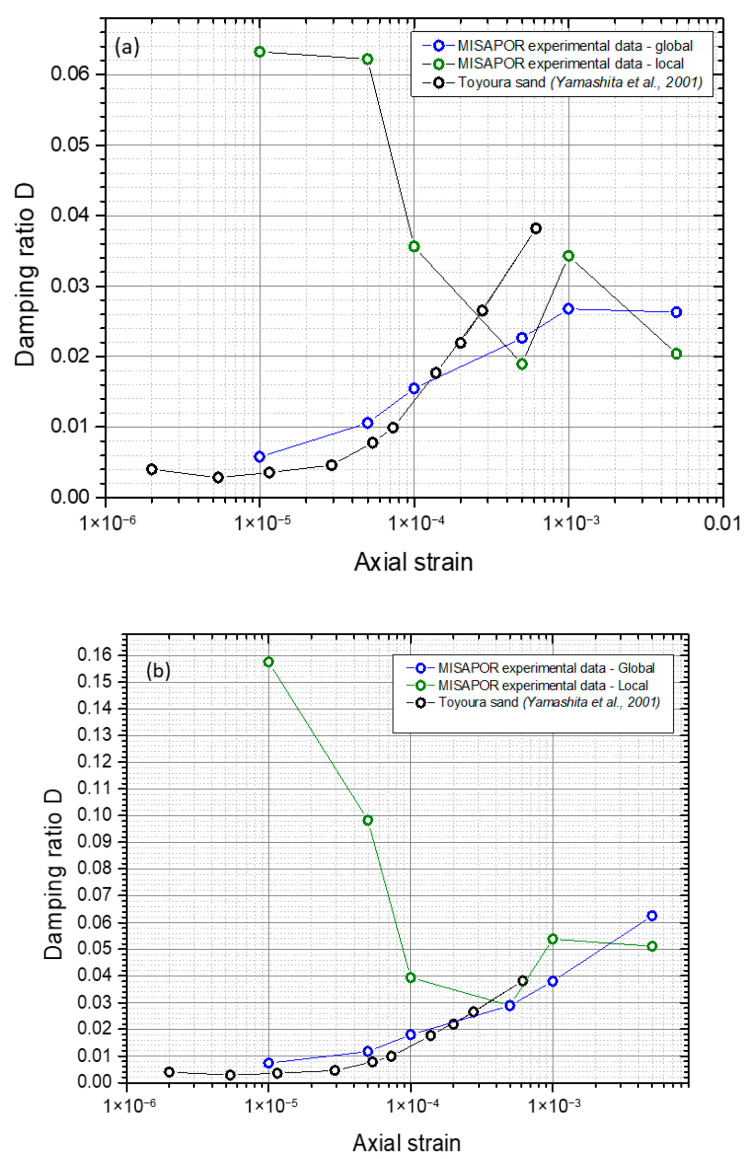
Variation in the damping ratio of CGA with the cyclic deformation level and comparison with Toyoura sand [44]: (**a**) raw data, and (**b**) estimation of the elastic energy based on the Young’s modulus.

As for the C7 test, the damping ratio was also measured for this test under an opposing pressure of around 100 kPa. The results of Figure 19 are compared to literature data for Toyoura sand. The curves are very close and nearly overlapping from the CGA and natural sand at relatively higher strains. Whereas, for smaller values of strains, a divergence in the curves is noted again, which can be explained by unclear loops for this level of strains. In terms of damping ratio, the CGA exhibits very similar behavior to that of natural sand.

This comparison with natural sands and gravelly soils is intended to be qualitative rather than one-to-one equivalence, as the difference in gradation curves, morphology and density state between CGA and natural sands may influence stiffness and damping ratio. Within these limitations, the results indicate that CGA exhibits damping behavior broadly similar to that of sand and gravelly soil under comparable stress and strain conditions.

**Figure 20:**
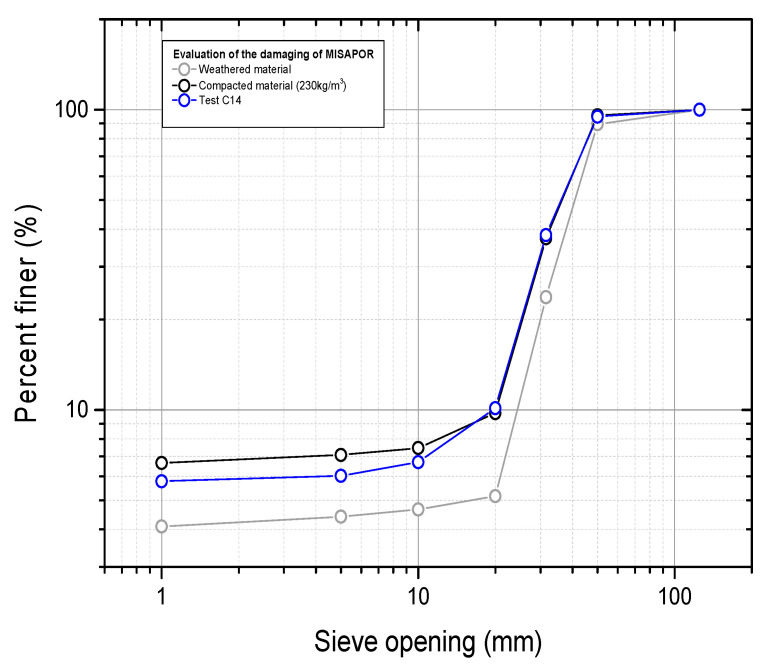
Evaluation of the damage of CGA caused by the cyclic test.

Figure 20 shows the material’s grading curve tested under six series of strains and confined at a pressure of 100 kPa. The graph also presents the grading curves corresponding to the weathered material and the compacted one. It can be noted that the curve approaches that of the compacted sample remarkably well, with a slight elevation in the fine fraction, which is expected to be a result of the crushing of medium-sized particles during compaction.

### 3.3. Effect of the Consolidation Stress

**Figure 21:**
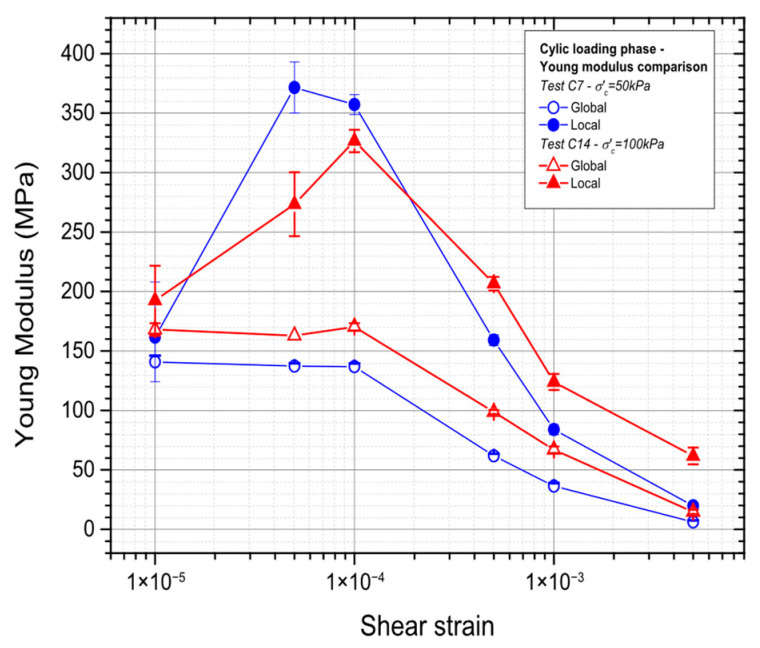
Evaluation of the influence of the consolidation stress on the Young’s modulus: global and local measurements.

The outcome of the Young’s modulus for the two consolidation stresses (50 kPa and 100 kPa) is depicted in Figure 21. It can be seen that as the value of consolidation stress increases, the values of Young’s modulus also increase for both local and global measurements. It is also noticed that for small strains, the curves diverge before they converge again for higher strains.

**Figure 22:**
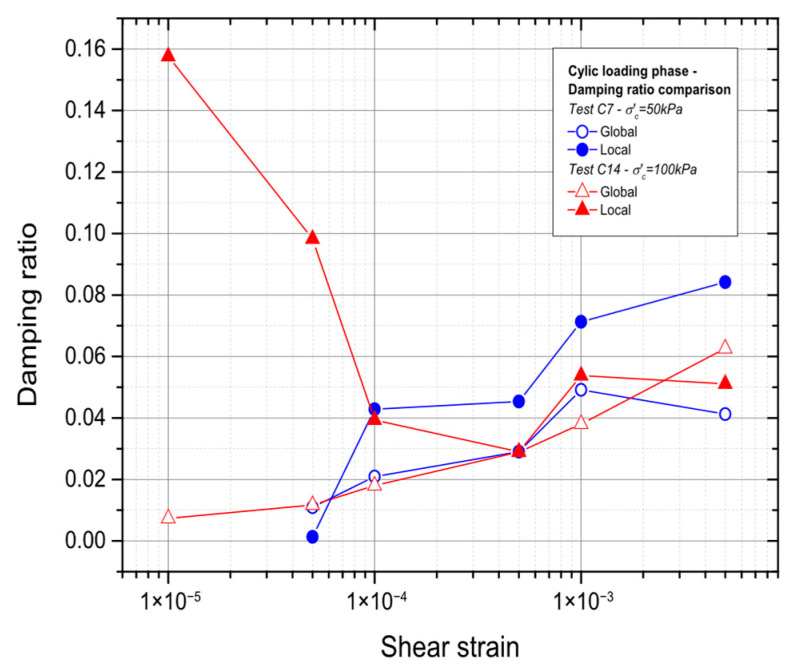
Evaluation of the influence of the consolidation stress on the damping ratio.

Additionally, the effect of the restricting pressure on the damping ratio was examined. Figure 22 displays the findings. Except for the first section, where the extremely tiny stresses show a divergence, the curves are nearly identical, and it is shown that the confining pressure does not affect the damping ratio.

## 4. Conclusions

This study systematically investigated the small to moderate strain cyclic response of cellular glass aggregates (CGA) under varying consolidation stresses. The experimental results provided insight into the stiffness and damping characteristics of CGA and its strain-dependent behavior. The principal findings are summarized below in accordance with the sequence of the research objectives addressed in the manuscript.

1. Environmental relevance and material potential: CGA demonstrates strong potential as a sustainable alternative to natural aggregates, contributing to reduced resource depletion and carbon emissions.
2. Cyclic behavior under different consolidation stresses: The cyclic response of CGA was evaluated under consolidation stresses of 50 kPa and 100 kPa, showing consistent trends in stiffness and damping behavior across both stress levels.
3. Small-strain stiffness characterization: At very small strain levels (in the order of 10^−5^), the evaluation of Young’s modulus was unreliable due to difficulties in clearly defining stress–strain hysteresis loops, highlighting the limitation in measurement accuracy at extremely low strains.
4. Behavior at higher strains: At higher strain amplitudes (10^−3^), CGA exhibited mechanical behavior comparable to that of gravelly soils, with nearly identical stiffness values, indicating its suitability for engineering applications.
5. Damping characteristics: Damping ratio measurements were affected by poorly defined hysteresis loop boundaries at low strain levels. Whereas at higher strains, the boundaries were clearly identifiable, resulting in more accurate and reliable damping estimates.
6. Applicability of small-strain measurements (10^−5^): Seismic parameters derived at very low strains (order of 10^−5^) cannot be considered reliable due to difficulty in defining the loops at these amplitudes. Consequently, these parameters are not suitable for quantitative interpretation and should not be taken into consideration. The seismic properties should be derived from strain levels for which well-defined loops and behavior is observed.
7. Comparison with natural soils: Overall, CGA showed very good agreement with natural soils (sand and gravelly soils) in terms of stiffness and damping behavior.

## Figures and Tables

**Table 1:** Experimental program.

Name	Class	(kPa) σ’c	(kg/m^3^) ρd	Accommodation	(ε_cyc_) Cyclic Loading	Number of Cycles
C7	Seismic	50	230	20,000 cycles q_cyc_ = 20 kPa	10^−5^	100 per step
					5 × 10^−5^	
					10^−4^	
					5 × 10^−4^	
					10^−3^	
					5 × 10^−3^	
C14	Seismic	100	230	20,000 cycles q_cyc_ = 40 kPa	10^−5^	100 per step
					5 × 10^−5^	
					10^−4^	
					5 × 10^−4^	
					10^−3^	
					5 × 10^−3^	

## Data Availability

The original contributions presented in this study are included in the article. Further inquiries can be directed to the corresponding author.

## Supplementary Materials

The following supporting information can be downloaded at: https://www.mdpi.com/article/10.3390/ma19050993/s1, Figure S1: Results for the rest deformation levels; Figure S2: Results for the rest deformation levels; Figure S3: Results for the rest deformation levels; Figure S4: Results for the rest deformation levels.
