# Supplementary material for: Cyclic Behavior of Cellular Glass Aggregates: An Experimental Comparison with Natural Aggregates
Source: Materials (Basel). 2026 Mar 4;19(5):993. doi: 10.3390/ma19050993 (PMC12986230; doi:10.3390/ma19050993)
Supplement: Supplementary file 1 [file materials-19-00993-s001.zip › materials-4079842-supplementary.pdf]

materials

Supplementary Material

Cyclic Behavior of Cellular Glass Aggregates: An Experimental Comparison with Natural Aggregates

Layal Jradi [1,*], Bassel Seif El Dine [1], Jean-Claude Dupla [2] and Jean Canou [2]

[1] College of Sciences and Human Studies, Prince Mohammad Bin Fahd University, Dhahran 34754, Saudi Arabia; bseifeldine@pmu.edu.sa
[2] Ecole des Ponts ParisTech, Champs sur Marne, 77455 Marne la Vallée CEDEX 2, France; jean-claude.dupla@enpc.fr (J.-C.D.)
* Correspondence: lseifeldine@pmu.edu.sa

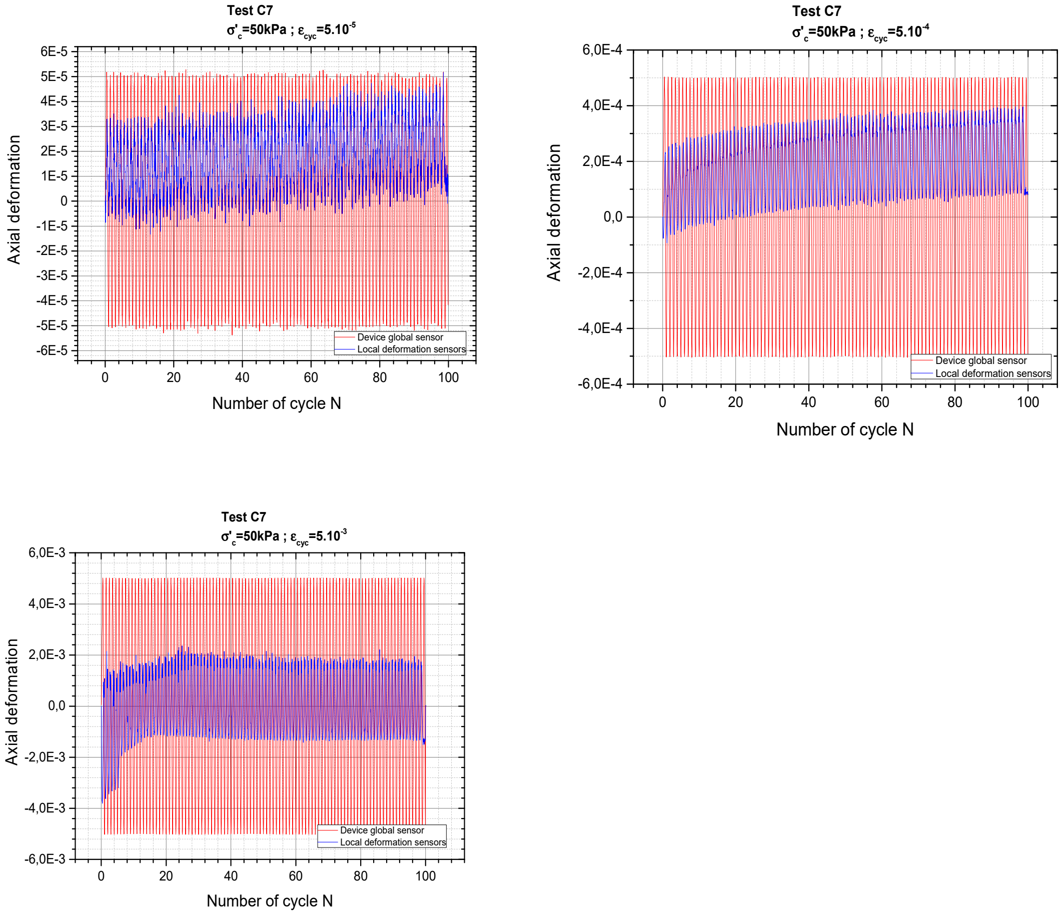

Figure S1. Results for the rest deformation levels.

 https://doi.org/10.3390/ma19050993

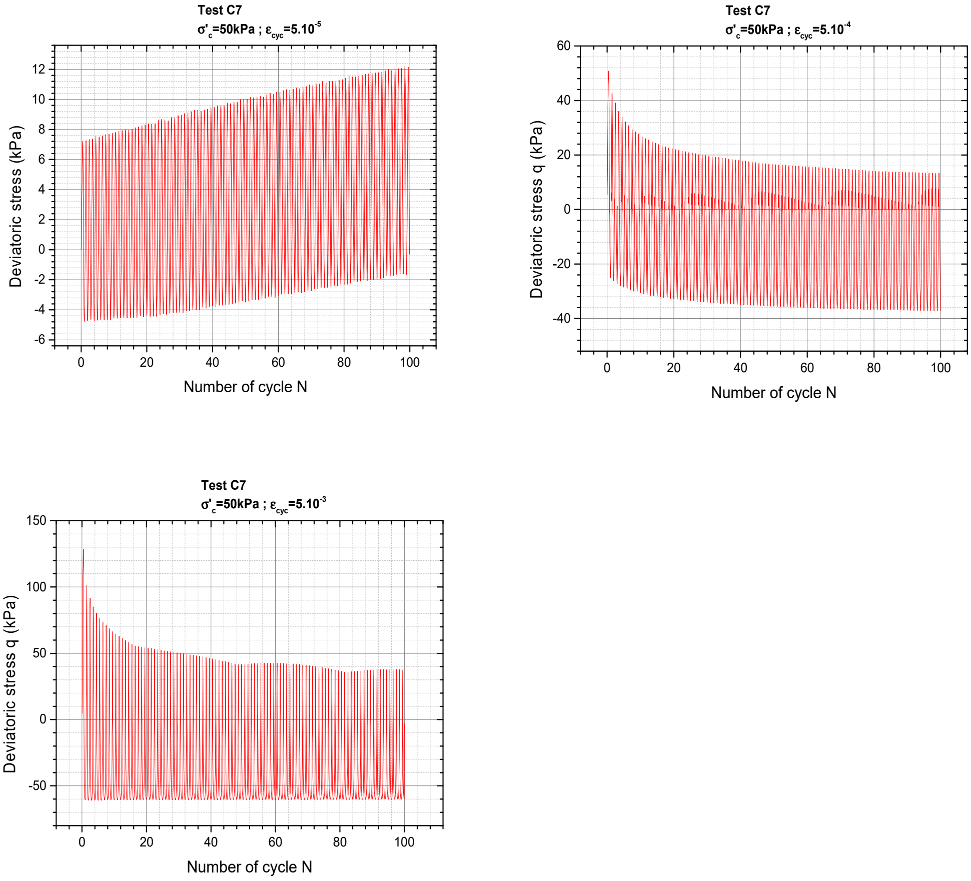

Figure S2. Results for the rest deformation levels.

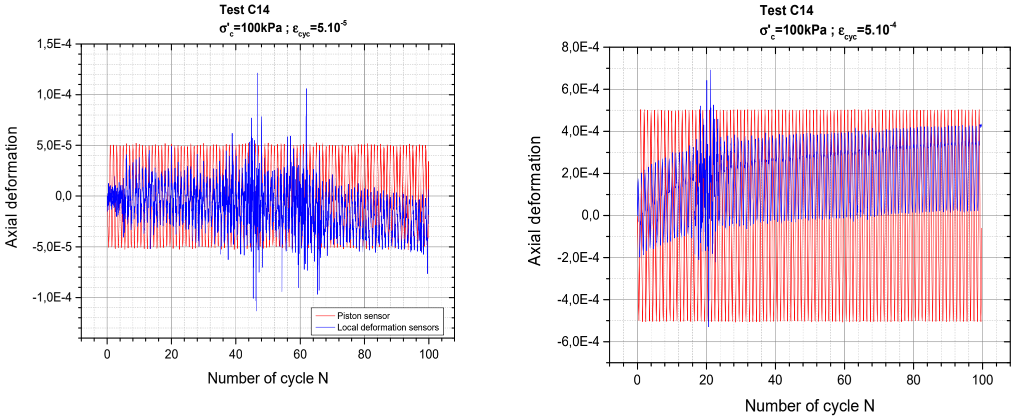

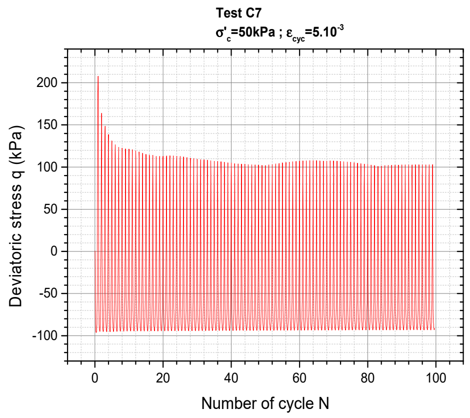

Figure S3. Results for the rest deformation levels.

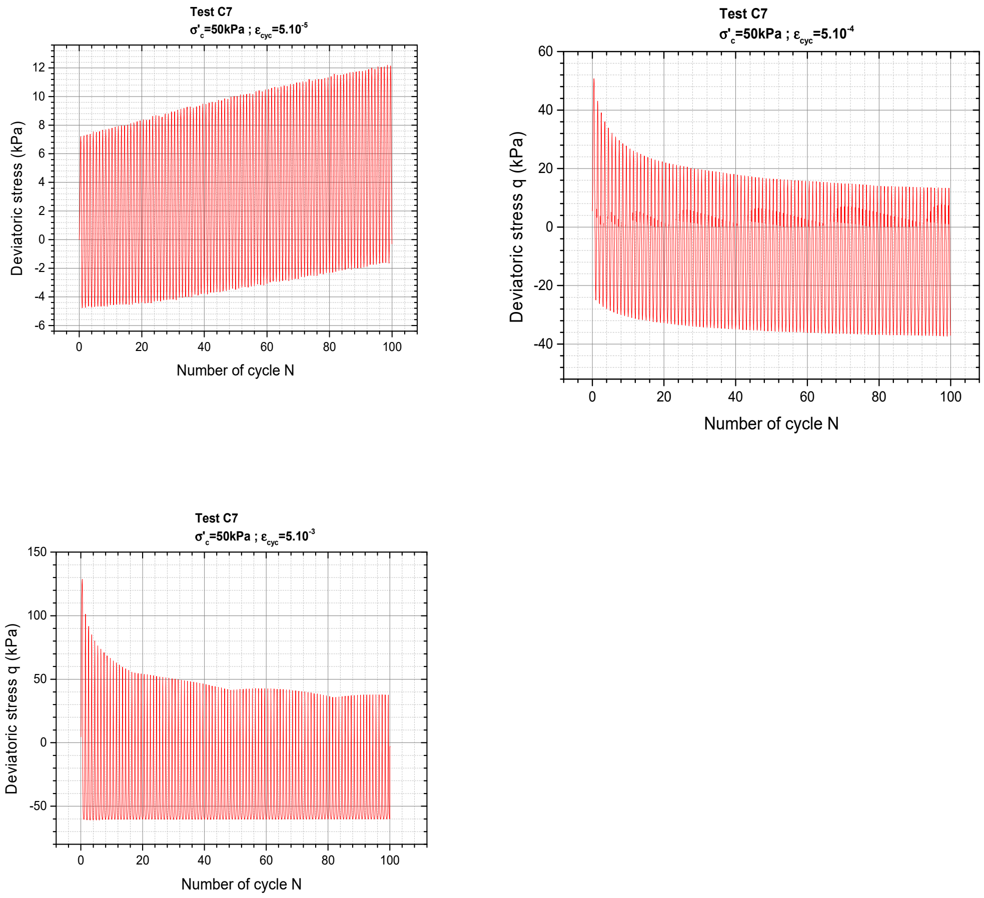

Figure S4. Results for the rest deformation levels.
